# The macular function and structure in patients with diabetic macular edema before and after ranibizumab treatment

**DOI:** 10.1007/s10633-016-9531-4

**Published:** 2016-03-21

**Authors:** Barbara Nowacka, Marta Kirkiewicz, Katarzyna Mozolewska-Piotrowska, Wojciech Lubiński

**Affiliations:** Department of Ophthalmology, Pomeranian Medical University, Powstancow Wlkp. 72, 70-111 Szczecin, Poland

**Keywords:** Diabetic macular edema (DME), Ranibizumab, OCT, PERG, mfERG

## Abstract

**Purpose:**

To evaluate macular function and structure in patients with diabetic macular edema prior to, as well as 3 and 6 months after intravitreal ranibizumab treatment.

**Patients and methods:**

Seventeen eyes of 17 patients with type 2 diabetes mellitus and diabetic macular edema (DME) were treated with intravitreal injections of 0.5 mg ranibizumab. Prior to the first injection, as well as after 3 and 6 months, the following examinations were performed: assessment of distance best-corrected visual acuity (log MAR), perception of metamorphopsia (M-Chart), slit lamp examination of the anterior and posterior segment of the eye (Volk 90D lens), evaluation of the retinal and choroidal circulation (fluorescein angiography), assessment of the structure and thickness of the macula (OCT), as well as evaluation of the macular function (PERG and mfERG).

**Results:**

We observed that ranibizumab significantly improved visual acuity after 3 and 6 months from the beginning of the treatment, which was a consequence of reduced macular edema and vascular leakage. There was a statistically significant decrease in metamorphopsia frequency at month 3; however, at month 6 it was a statistically insignificant when compared to the baseline. The results of electrophysiological examinations revealed no improvement in ranibizumab-treated patients.

**Conclusion:**

Improvement of visual acuity and reduction in macular thickness were maintained up to the 6-month follow-up. The results of electrophysiological examinations revealed that ranibizumab injections tend to stabilize bioelectrical macular function of the outer, middle and inner retinal layers, which was impossible to recognize on the basis of visual acuity and OCT. Therefore, the electrophysiological examinations should be used as an additional objective tool for the evaluation of the anti-VEGF treatment effectiveness in DME.

## Introduction

Diabetic retinopathy (DR) is a leading cause of vision loss in working-age patients around the world, and diabetic macular edema (DME) is its major vision-threatening complication [1–3]. The results of many studies indicate that one-fourth of patients with DR suffer from DME [4]. Therefore, with increasing number of new cases of type 2 diabetes each year, DME may become a significant public health issue. The results of the Early Treatment Diabetic Retinopathy Study (ETDRS) demonstrated that focal/grid photocoagulation of DME might reduce the risk of moderate visual loss by approximately 50 % and it has been established as the gold standard treatment [5]. However, there are many cases where it carries a significant risk, or efficient laser treatment could not be performed due to diffuse macula edema. In the aqueous humor of diabetics with DME, increased level of the vascular endothelial growth factor (VEGF)—a significant blood–retinal barrier breakdown mediator—was observed, which led to the hypothesis that alternative or adjunct therapies using VEGF inhibitors could be beneficial in reversing vision loss from macular edema [6, 7]. The current standard methods for evaluating the effectiveness of the anti-VEGF treatment are visual acuity (VA) and optical coherence tomography (OCT). Visual acuity indirectly provides information about foveal function, while OCT reveals only structural changes of the macula. That is why we decided to also provide information about retinal and choroidal circulation abnormalities (fluorescein angiography—FA) and the macular function (pattern and multifocal electroretinograms—PERG and mfERG) before and after intravitreal anti-VEGF treatment. The results of the electrophysiological examinations contribute comprehensive information about macular function in the course of DME because PERG explores mostly ganglion cells’ function (the inner layer of the retina), while mfERG explores bipolar cells’ and cone photoreceptors’ (the middle and outer layers). Moreover, the electrophysiological examinations provide greater information about the macular function, while VA corresponds to only 1 degree field of vision. According to our best knowledge, there are only 2 studies in the literature describing PERG/mfERG recordings in the DME eyes treated with ranibizumab [8, 9] and our study for the first time illustrates inner and outer macular function changes before, as well as 3 and 6 months after the beginning of intravitreal treatment.

## Patients and methods

Seventeen eyes of 17 patients (8 males, 9 females) aged 65 ± 10 years with type 2 diabetes mellitus and DME were treated with intravitreal injections of 0.5 mg ranibizumab. The mean time of diabetes mellitus was 17 ± 10.5 years. At baseline, the mean hemoglobin A1C ± SD was 7.56 % ± 1.94. Exclusion criteria were as follows: focal/grid laser treatment within 3 months, intraocular injection of steroid within 3 months, intraocular injection of a VEGF antagonist within 2 months, eccentric fixation, bad cooperation and potential contributing causes to reduced macular function other than DME, for example glaucoma or significant cataract. The first 3 intravitreal injections of 0.5 mg/0.1 ml ranibizumab were obligatory performed every 4 weeks. The decisions about additional injections were made up to 6 months. The therapy was not continued when the thickness of the fovea reached ≤225 μm and BCVA ≥79 letters, and was restarted when the thickness of the fovea increased of at least 50 μm or was a BCVA reduction in 5 letters and drop below 74 letters. Prior to the first injection, as well as after 3 and 6 months, the following examinations were performed: assessment of distance best-corrected visual acuity (DBCVA) (log MAR), perception of metamorphopsia (M-Chart), slit lamp examination of the anterior and posterior segment of the eye (Volk 90D lens), evaluation of the retinal and choroidal circulation (FA), assessment of the structure and thickness of the macula (Cirrus HD-OCT, Carl Zeiss Meditec, Dublin, CA, USA), as well as evaluation of the macular function—PERG and mfERG (Roland Consult, Germany).

All parameters of PERG and mfERG were consistent with the current International Society for Clinical Electrophysiology of Vision (ISCEV) Standards [10, 11]. The electrodes used for recordings were as follows: active electrode—thread DTL electrode positioned above the upper margin of the lower eyelid in contact with the cornea, and reference electrode—gold disc electrode placed on the skin near the ipsilateral outer canthus of the examined eye and ground gold disc electrode placed on the forehead (Fpz). The acceptable electrode impedances were below 5 kΩ.

### Pattern electroretinogram

The examination was performed with appropriate optical correction for a distance of 0.5 meters and without pupil dilatation, which ensured good retinal image quality. During the examination, monocular stimulation and central fixation were used. Stimulus parameters were as follows: black and white reversing checkerboard with a check size of 1°, luminance of the white elements of 120 cd/m^2^, and contrast between black and white squares of 97 %. Parameters of the recording system were as follows: amplifiers sensitivity: 20 µV/div; filters: 1–100 Hz; notch filters: off; sweep time: 250 ms (time base: 25 ms/div); and artifact reject threshold: 25 % (for the amplifiers range ±100 µV). Two trials of 100 artifact-free sweeps for each eye were obtained and averaged off-line. The analysis included measurements of the amplitude and the peak time of P50 wave, the amplitude of N95 wave, as well as N95/P50 ratio.

### Multifocal electroretinogram

The examination was performed with appropriate optical correction for a distance of 30 cm, and patient’s pupils were maximally dilated (>6 mm) with 10 % Neosynephrine. During the examination, monocular stimulation and central fixation were used. Stimulus parameters were as follows: a black and white matrix of 61 scaled hexagons (distortion factor equal to 4) displayed in the 50° field of vision, luminance for white elements of 100 cd/m^2^, and the contrast between black and white hexagons of 97 %. Parameters of the recording system were as follows: amplifiers sensitivity: 20 µV/div; filters: 10–300 Hz; notch filters: off; plots time: 83 ms; and artifact reject threshold: 8 % (for the amplifiers range ±100 µV). Six cycles were averaged off-line including digital smoothing (2×), software reduction in line interference and manual correction, if necessary, applied to the automatic cursors placement. The analysis included response density (the response amplitude divided by the retinal area—nV/deg^2^) and peak time of the P1-wave in ring 1 (R1) and ring 2 (R2), which correspond to the foveal and parafoveal retinal area, respectively.

All subjects participating in this study gave their informed written consent. The project was approved by the Ethics Committee of the Pomeranian Medical University.

The Shapiro–Wilk test was used to evaluate the normality of distribution of analyzed parameters. The Student *t* test was used for the normal and Wilcoxon signed-rank test for non-normal distributed data. To address the problem of multiple comparisons, the false discovery rate (FDR) methodology was used. Corrected probabilities are presented in the manuscript. The *p* value <0.05 was considered as significant.

## Results

During 6-month follow-up time, 47.1 % (8/17) eyes required only 3 consecutive ranibizumab injections, 35.3 % (6/17) eyes required 4 injections, and 17.6 % (3/17) eyes required 5 injections.

The progression from the non-proliferative to the proliferative retinopathy occurred in 1 eye (5.6 %) despite appropriate anti-VEGF therapy at this time.

### Distance best-corrected visual acuity

**Fig. 1:**
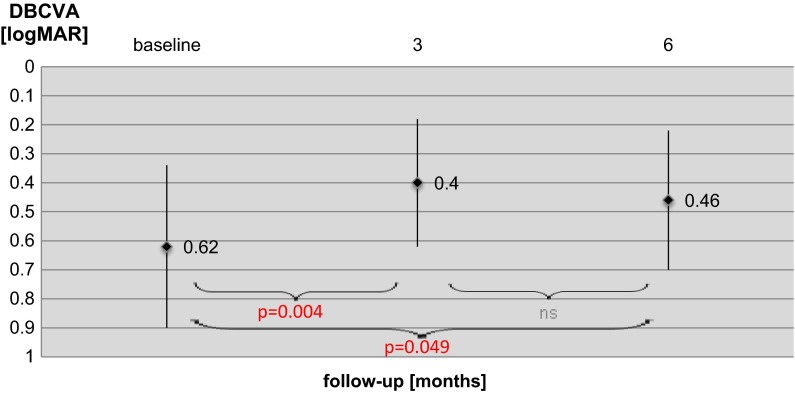
Results of DBCVA in eyes of patients with DME at the baseline, 3 and 6 months after beginning treatment with intravitreal ranibizumab injections. Data are presented as mean and standard deviation. *ns* not significant (*p* > 0.05)

The mean DBCVA at the baseline was equal to 0.62 ± 0.28 (log MAR scale) and improved significantly to 0.4 ± 0.22 (*p* = 0.004) after 3 months. Between third and sixth month DBCVA slightly decreased to 0.46 ± 0.24, but this result was statistical insignificant compared to mean DBCVA at month 3. However, after 6 months from the baseline improvement DBCVA was still statistically significant compared to baseline (*p* = 0.049). The results of DBCVA examinations are presented in Fig. 1.

### Perception of metamorphopsia

**Fig. 2:**
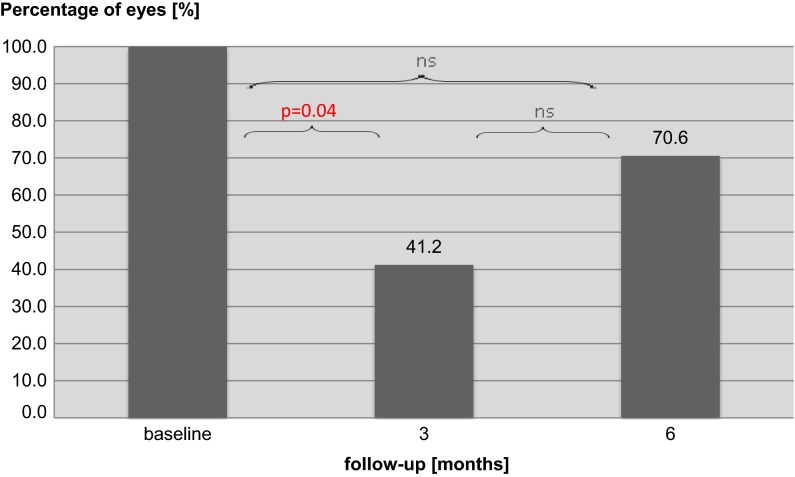
Percentage of patients reporting metamorphopsia at the baseline, 3 and 6 months after beginning treatment with intravitreal ranibizumab injections. *ns* not significant (*p* > 0.05)

At the baseline, 15 from 17 (88.2 %) patients complained of metamorphopsia in the eye recruited to intravitreal ranibizumab treatment. Distribution of metamorphopsia according to M-Chart examination was as follows: 10 patients—0°, 1 patient—0.3°, 1 patient—0.4°, 2 patients—1.1° and 1 patient—2°. After 3-month follow-up, only 7 patients (41.2 %) reported metamorphopsia and distribution of metamorphopsia was: 5 patients—0° and 2 patients—0.2°. The result was statistically significant (*p* = 0.04). However, after 6 months the number of patients with metamorphopsia increased to 12 (70.6 %), which was statistically insignificant in comparison with the baseline (*p* > 0.05). Distribution of metamorphopsia after 6 months from the beginning of the intravitreal treatment was as follows: 9 patients—0°, 2 patients—0.3° and 1 patient—0.6°. Insightful analysis revealed that 2 patients, who did not report metamorphopsia at the baseline, had significant macular edema (the mean foveal thickness was 705.5 µm, and the parafoveal thickness was 572 µm in OCT) and their DBCVA was almost the same as in the metamorphopsia present group (logMAR 0.63 vs. 0.62). This might suggest that all patients at the baseline reported metamorphopsia, but these 2 patients did not understand or do not perform the M-Chart examination properly due to the low quality of vision. For this reason, it may be assumed that all patients reported metamorphopsia at the baseline. Figure 2 presents the percentage of patients reporting metamorphopsia at 6-month follow-up. Statistical analysis of the relationship between metamorphopsia and the other tests’ results was performed at third and sixth month. As it was expected, after 3- and 6-month follow-up the non-metamorphopsia group had better DBCVA P50 and N95 amplitudes of PERG, as well as the mean P1-response density in R1 and R2 in mfERG. However, statistical analysis has limited value due to small groups of patients in comparison. Therefore, these data present only a tendency and they are summarized in Table 1.

**Table 1:** The relationship between perception of metamorphopsia and visual acuity, OCT and electrophysiological tests’ (PERG and mfERG)

	M (−) group	M (+) group	*p*
3rd month			
DBCVA	0.37	0.45	ns
OCT			
FT	314.6	339.3	ns
PFT	353.4	379.8	ns
PERG			
A P50	2.2	1.7	ns
A N95	3.2	2.6	ns
mfERG			
P1–R1	44.4	32.3	ns
P1–R2	21.0	14.4	ns
6th month			
DBCVA	0.37	0.50	ns
OCT			
FT	338.5	390.0	ns
PFT	379.0	405.3	ns
PERG			
A P50	2.2	1.7	ns
A N95	2.9	2.5	ns
mfERG			
P1–R1	40.4	37.5	ns
P1–R2	18.5	12.2	ns

The results are presented as a mean value

*M (−) group* patients without metamorphopsia, *M (+) group* patients with metamorphopsia, *DBCVA* distance best-corrected visual acuity, *OCT* optical coherence tomography, *FT* foveal thickness, *PFT* parafoveal thickness, *PERG* pattern electroretinogram, *A P50* P50 wave amplitude, *A N95* N95 wave amplitude, *mfERG* multifocal electroretinogram, *P1−R1* P1-response density in ring 1, *P1−R2* P1-response density in ring 2

### Optical coherence tomography

**Fig. 3:**
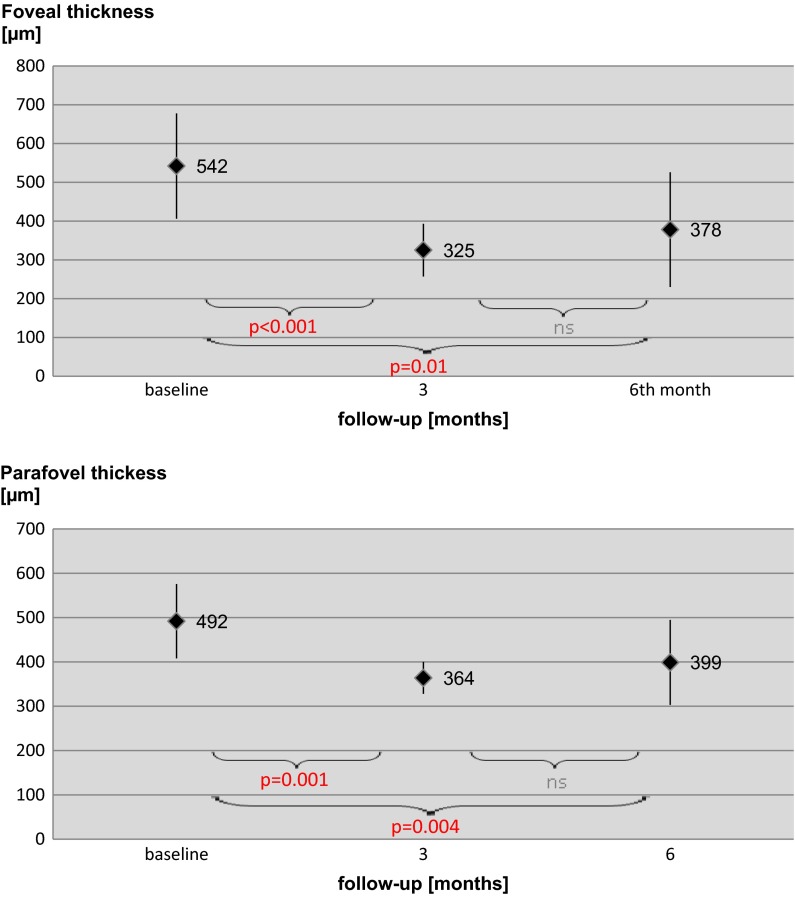
Results of OCT examinations in eyes of patients with DME at the baseline, 3 and 6 months after beginning treatment with intravitreal ranibizumab injections. Data are presented as mean and standard deviation. *ns* not significant (*p* > 0.05)

After 3 months from the first ranibizumab injection, there was a significant decrease in the mean foveal thickness (FT) from 542 ± 136 µm to 325 ± 68 µm (*p* < 0.001). After 6 months, mean FT slightly increased to 378 ± 148 µm compared to result in the third month, but this was statistically insignificant (*p* = 0.12). However, after 6 months decrease in the mean FT was still statistically significant compared to baseline (*p* = 0.01). The results of the parafoveal thickness were changing similarly to FT—the baseline: 492 ± 84 µm, third month: 364 ± 36 µm (*p* = 0.001), sixth month: 399 ± 96 µm (*p* = 0.004 compared to the baseline). The results of foveal and parafoveal thickness during 6-month follow-up are presented in Fig. 3.

### Fluorescein angiography (FA)

**Fig. 4:**
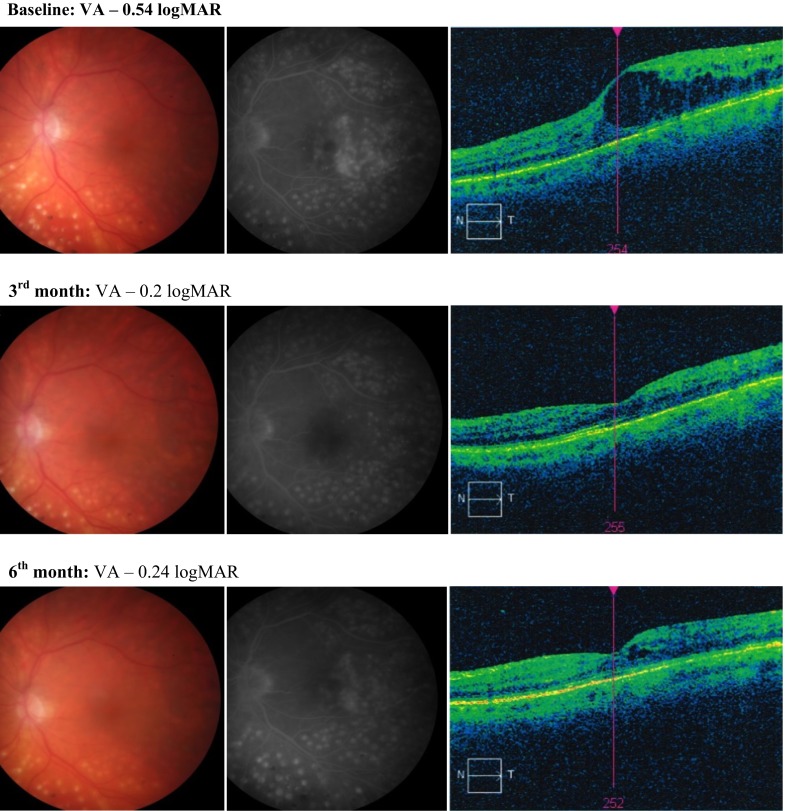
Examples of the fundus color photography, FA and OCT results of the patient’s eye with DME at the baseline, as well as at the 3- and 6-month follow-up. At baseline, FA revealed a massive leakage of dye, corresponding to the diabetic macular edema seen on OCT scan and color photography. After 3 months of treatment, no dye leakage was visible in macular region and OCT scan showed reduction in macular thickness. However, after 6 months of follow-up an increase in dye leakage in FA and macular thickness in OCT was revealed

At baseline, a massive leakage of dye, corresponding to the diabetic macular edema, was seen in 100 % (17/17) eyes. AF results 4 weeks after third anti-VEGF injection showed no dye leakage only in 2 eyes (11.8 %) and reduced the intensity or the area covered by leakage of dye in 4 eyes (23.5 %). In the other eyes, despite improvement of DBCVA and reduction in the macular thickness in OCT, the image of a leak in FA remained unchanged. Similar results were obtained in FA performed 6 months after initiation of anti-VEGF therapy, in which no evidence of leakage of dye in the macular area was visible in 3 eyes (17.7 %) and a significant reduction in the intensity or the area covered by a leakage in 4 eyes (23.5 %). The examples of the fundus color photography, FA and OCT results of the patient’s eye with DME at the baseline as well as at the 3- and 6-month follow-up are presented in Fig. 4.

### Pattern electroretinogram

**Fig. 5:**
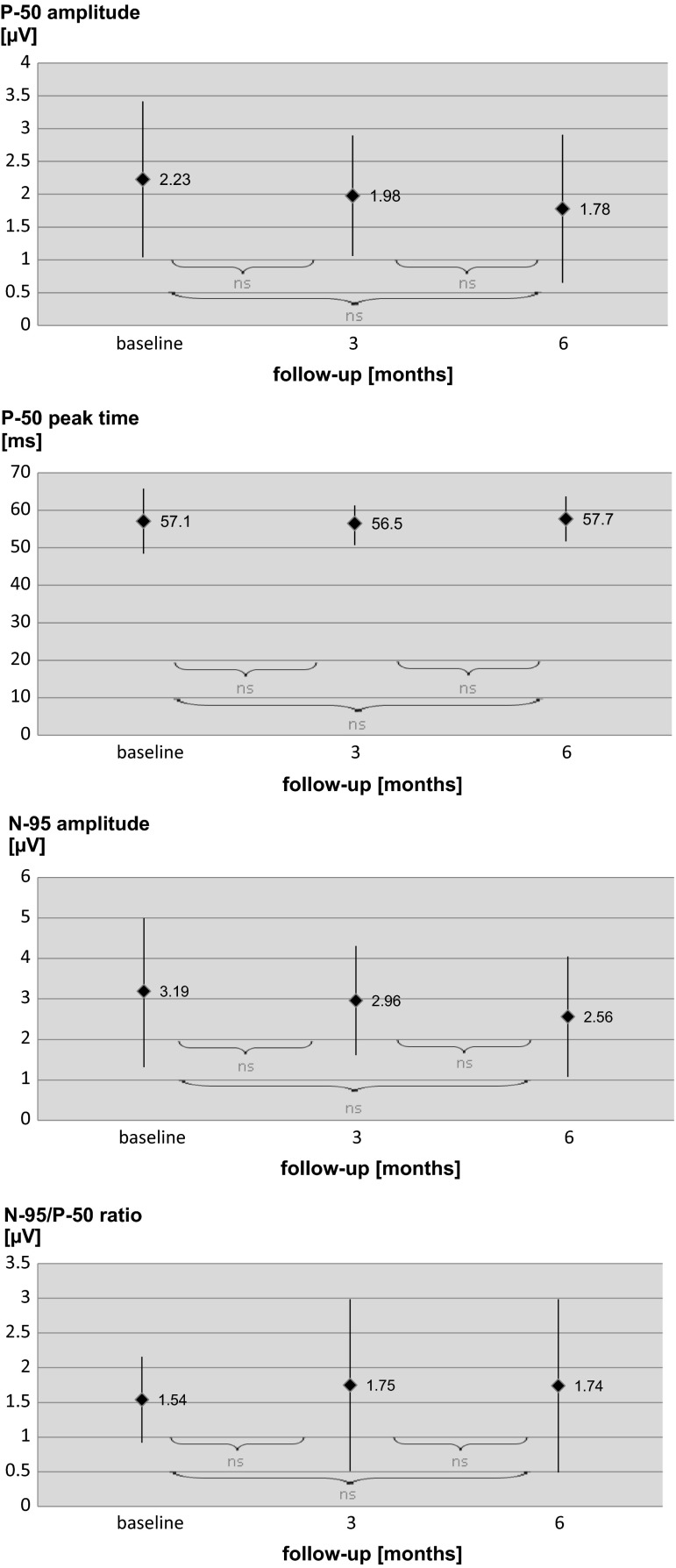
Results of the PERG obtained in eyes of patients with DME at the baseline, 3 and 6 months after beginning treatment with intravitreal ranibizumab injections. Data are presented as mean and standard deviation. *ns* not significant (*p* > 0.05)

The mean P50 amplitude at the baseline was equal to 2.23 ± 1.19 µV and slightly decreased after 3 (1.98 ± 0.92 µV, *p* > 0.05) and 6 months (1.78 ± 1.13 µV, *p* > 0.05). The mean P50 peak time was equal to 57.1 ± 8.7 ms and remained almost unchanged during the whole examinations of the follow-up (56.5 ± 5.8 ms after third month, 57.7 ± 6.0 ms after the sixth month, *p* > 0.05). After 3- and 6-month follow-ups, the mean P50 amplitude, as well as the mean P50 peak time, did not significantly differ in comparison with the baseline. The mean N95 amplitude changed similarly to P50 amplitude—the baseline: 3.19 ± 1.81 µV, third month: 2.96 ± 1.35 µm (*p* > 0.05), sixth month: 2.56 ± 1.49 µm (*p* > 0.05 compared to the baseline). Also, N95/P50 amplitude ratio did not significantly change during the follow-up. The results of the PERG are summarized in Fig. 5. At baseline, 14/17 eyes demonstrated reduced amplitudes of P50 and N95 waves in comparison with norms defined in our laboratory for patients above 50 years old as mean ± 2SD (P50 = 3.2–11.3 µV; N95 = 4.8–15.7 µV). On this basis, we decided to divide retrospectively patients into two groups: first with normal PERG and second with abnormal PERG. The level of HbA1c was almost the same in normal and abnormal PERG group (7.2 vs. 7.6 mg %, respectively). In the normal PERG group, the baseline visual acuity was better than in the abnormal PERG group (0.52 vs. 0.64 logMAR), but foveal and parafoveal thicknesses had similar values (551 vs. 540 and 500 vs. 491 µm, respectively). After the 3-month follow-up, the normal PERG group gained on average 15 letters on ETDRS chart, while the abnormal PERG group only 10. At this point of time, the foveal thickness was also lower in the normal PERG group in comparison with the abnormal PERG group (275 vs. 335 µm), while parafoveal thickness was almost the same like in both groups group (371 vs. 363 µm). After the 6-month follow-up, the normal PERG group VA was still 15 letters better in comparison with the baseline, while the abnormal PERG group maintained a result of only 6 letters better visual acuity. At the end point of the study, the foveal thickness was also better in the normal PERG group in comparison with the abnormal PERG group (342 vs. 386 µm), while parafoveal thickness was almost the same in both groups (401 vs. 399 µm). On the basis of these results, it is reasonable to suppose that normal PERG results prior to ranibizumab treatment may be an indicator for its better 6-month effectiveness. However, further research is needed to confirm this tendency.

### Multifocal electroretinogram

**Fig. 6:**
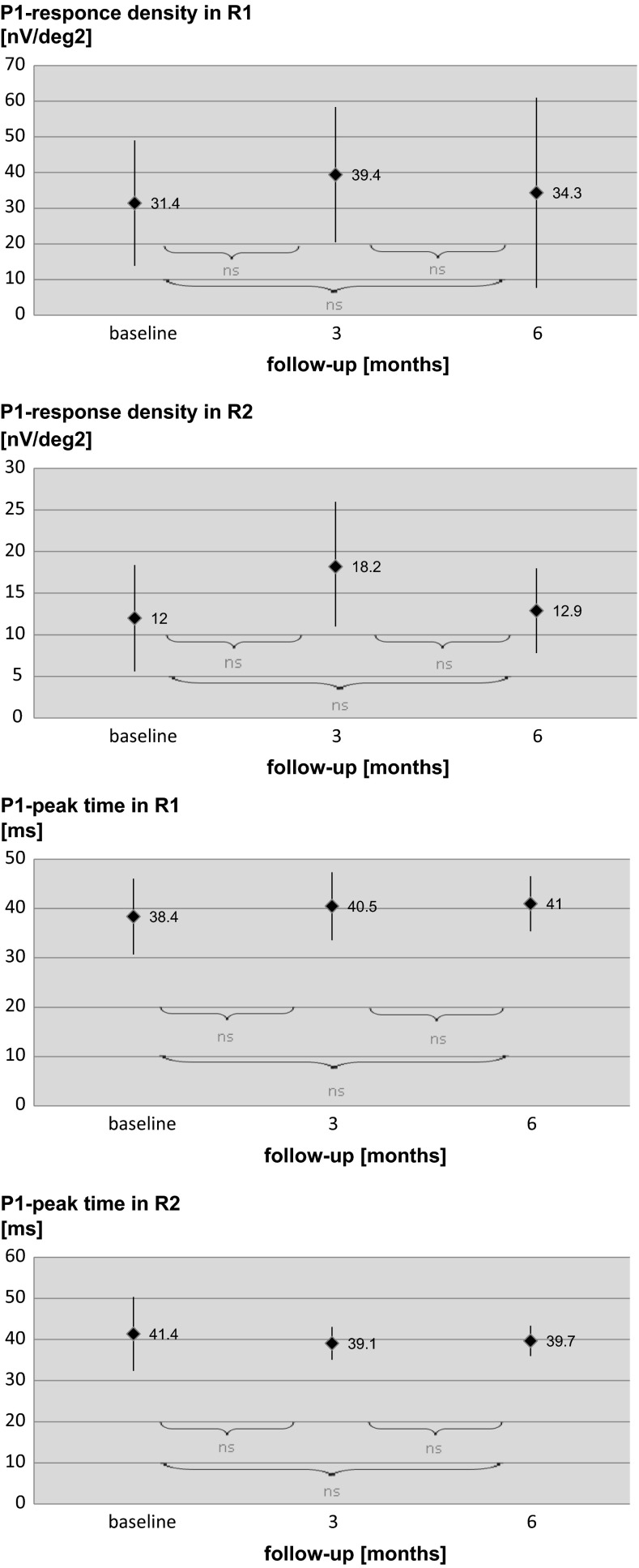
Results of mfERG obtained in eyes of patients with DME at baseline and 3 and 6 months after beginning treatment with intravitreal ranibizumab injections. *ns* not significant (*p* > 0.05)

**Fig. 7:**
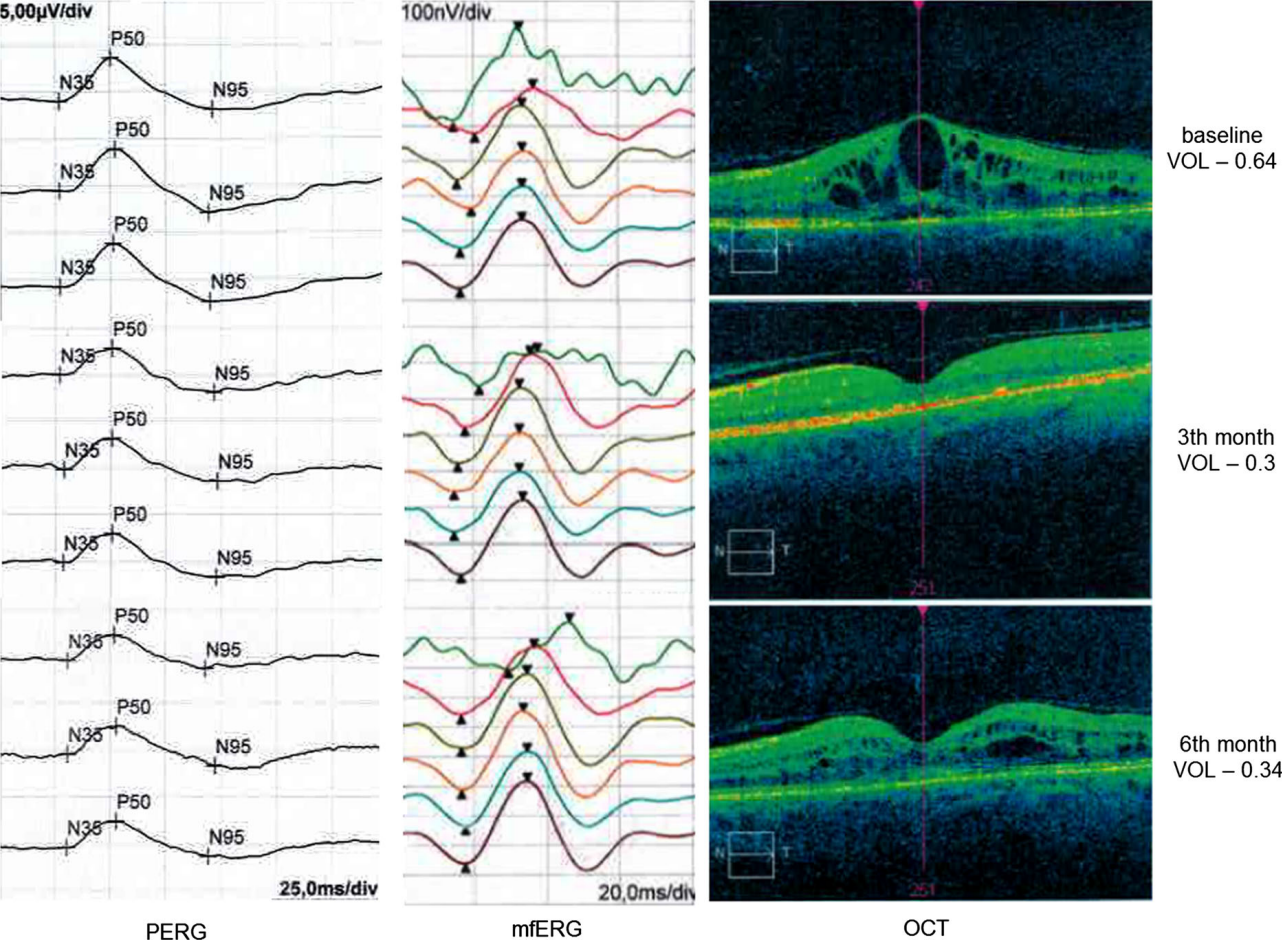
Example of PERG and mfERG results from the one eye of one patient in comparison with OCT and VA during the 6-month follow-up. The results of electrophysiological examinations showed no improvement—with the exception of the increased mean P1-response density in R2. The visual acuity after 3 and 6 months from the beginning of the treatment was improved, which was a consequence of reduced macular edema observed in OCT

The mean P1-response density in R1 was equal to 31.4 ± 17.6 µV. After 3-month follow-up, it increased to 39.4 ± 19.0 µV; however, the difference was statistically insignificant. Instead of intravitreal ranibizumab treatment, the mean P1-response density in R1 at month 6 was almost the same as at the baseline (34.3 ± 26.7 µV, *p* > 0.05). The mean P1-response density in R2 at the baseline was equal to 12.0 ± 6.4 µV. In the third month, this density slightly increased to 18.2 ± 7.8 µV (*p* > 0.05). However, as P1-response density in R1, it decreased to almost the same value as at the baseline after 6-month follow-up (12.9 ± 5.1 µV, *p* > 0.05). After 3- and 6-month follow-up, the mean P1-peak time (R1 and R2) also did not differ significantly in comparison with the baseline. The results of the mfERG are summarized in Fig. 6. The example of PERG and mfERG results from the one eye of one patient in comparison with OCT and VA during the 6-month follow-up is shown in Fig. 7. At baseline, 15/17 eyes demonstrated reduced P1 amplitudes’ densities in R1 and R2 in comparison with norms defined in our laboratory for patients above 50 years old as mean ± 2SD (P1 = 62.27–130.89 nV/deg^2^; P2 = 30.21–72.24 nV/deg^2^). Dividing the patients into two groups (normal and abnormal mfERG) revealed that patients with P1 amplitude within normal limits had much better DBCVA (0.44 logMAR) and foveal/parafoveal thicknesses (403/431 µm) than patients with abnormal mfERG results (0.64 logMAR, 560/500 µm, respectively). Although normal mfERG group gained fewer letters after 3 months of ranibizumab therapy in comparison with the abnormal mfERG group (5 vs. 12 letters), their DBCVA was slightly better at third month (0.34 vs. 0.40) and more stable in comparison with the abnormal mfERG group (0.36 vs. 0.48 at sixth month). The results of foveal/parafoveal thicknesses changed similarly to DBCVA results, with greater reduction in the abnormal mfERG group in the third month, but clearer stabilization between third and sixth month in normal mfERG group. Similarly to PERG results, mfERG examinations showed a tendency for better response to intravitreal ranibizumab treatment in eyes with normal P1 amplitudes’ densities in R1 and R2 at baseline, but further research on the higher amount of eyes is needed to confirm these findings.

## Discussion

According to our best knowledge, the present study for the first time described many aspects of the intravitreal ranibizumab treatment effectiveness. We observed that ranibizumab significantly improved visual acuity after 3 and 6 months from the beginning of the treatment, which was a consequence of reduced macular edema and vascular leakage. The results of previous studies concerning the relationship between visual acuity or macular thickness and intravitreal ranibizumab treatment confirm our findings [8, 12–15] even though there were some differences in frequency of ranibizumab injections and macular photocoagulation was applied if eligible. We also observed that there was a statistically significant decrease in metamorphopsia frequency at month 3 in ranibizumab-treated patients. However, after 6 months of intravitreal ranibizumab treatment, the frequency of metamorphopsia was statistically insignificant compared to baseline. This is probably a result of increased foveal and parafoveal thickness, which even though was significantly reduced at month 6 when compared to baseline, it was still outside the normal limits. In the available literature, we did not find any reports about the relationship between intravitreal ranibizumab treatment and AF results. Although ranibizumab seems to seal a blood–retinal barrier, we observed reduction or no dye leakage in less than half of treated eyes. The results of electrophysiological examinations (PERG and mfERG) revealed no improvement in ranibizumab-treated patients. The mfERG stimuli location and anatomic area of R1 (0.0–2.3°) corresponded roughly to the fovea and of R2 (2.3–7.4°) to the parafovea and partially to the perifovea. The difference in response in the fovea and parafovea might be a result of the predominance of the functional over structural changes in the latter. The decrease in the macular edema resulted in the enhancement of synaptic connectivity at month 3. Unfortunately, this positive effect was not maintained at month 6, which was probably a result of macular edema increase detected in OCT. The latest mfERG study of Holm et al. [8] also did not reveal improvement of macular function 3 months after beginning of the intravitreal treatment. Although PERG is widely used as a macular function index, its visual stimulus activates large retinal area. Probably as a result of the lower sensitivity of small areas function change, we did not observe any significant changes in PERG responses in contrast to mfERG. Up to date, we found only one study in the literature [9], which has evaluated functional effects of ranibizumab therapy with both electrophysiological examinations—PERG and mfERG. Consistently with our results, Comyn et al. [9] observed a small decrease in P50 amplitude in months 3 and 6, whereas P50 peak time remained almost unchanged. Also, N95 amplitude did not change significantly during follow-up. Although authors also performed mfERG in ranibizumab-treated patients, results are presented only after 1 year of treatment, which make a comparison with our results not possible.

## Conclusion

In the present study, improvement in visual acuity and reduction in macular thickness were maintained up to 6-month follow-up. However, results of electrophysiological examinations indicated on the significant and persistent dysfunction of the macula in our patients with DME. They revealed that ranibizumab injections tend to stabilize the bioelectrical macular function of the outer, middle and inner retinal layers, which is impossible to be recognizing on the basis of VA and OCT. Therefore, the electrophysiological examinations should be used as an additional objective tool for the evaluation of the anti-VEGF treatment effectiveness in DME.
